# Modelling Fungal Growth, Mycotoxin Production and Release in Grana Cheese

**DOI:** 10.3390/microorganisms8010069

**Published:** 2020-01-02

**Authors:** Marco Camardo Leggieri, Amedeo Pietri, Paola Battilani

**Affiliations:** 1 Department of Sustinable Crop Production, Università Cattolica del Sacro Cuore, Via E. Parmense, 84, 29122 Piacenza, Italy; marco.camardoleggieri@unicatt.it; 2 Department of Animal Science, Food and Nutrition, Università Cattolica del Sacro Cuore, Via E. Parmense, 84, 29122 Piacenza, Italy; amedeo.pietri@unicatt.it

**Keywords:** ochratoxin A, sterigmatocystin, *Penicillium*, *Aspergillus versicolor*, temperature, ecology, modelling, partitioning

## Abstract

No information is available in the literature about the influence of temperature (T) on *Penicillium* and *Aspergillus* spp. growth and mycotoxin production on cheese rinds. The aim of this work was to: (i) study fungal ecology on cheese in terms of T requirements, focusing on the partitioning of mycotoxins between the rind and mycelium; and (ii) validate predictive models previously developed by in vitro trials. Grana cheese rind blocks were inoculated with *A. versicolor*, *P. crustosum*, *P. nordicum*, *P. roqueforti,* and *P. verrucosum*, incubated at different T regimes (10–30 °C, step 5 °C) and after 14 days the production of mycotoxins (ochratoxin A (OTA); sterigmatocystin (STC); roquefortine C (ROQ-C), mycophenolic acid (MPA), Pr toxin (PR-Tox), citrinin (CIT), cyclopiazonic acid (CPA)) was quantified. All the fungi grew optimally around 15–25 °C and produced the expected mycotoxins (except MPA, Pr-Tox, and CIT). The majority of the mycotoxins produced remained in the mycelium (~90%) in three out of five fungal species (*P. crustosum*, *P. nordicum,* and *P. roqueforti*); the opposite occurred for *A. versicolor* and *P. verrucosum* with 71% and 58% of STC and OTA detected in cheese rind, respectively. Available predictive models fitted fungal growth on the cheese rind well, but validation was not possible for mycotoxins because they were produced in a very narrow T range.

## 1. Introduction

Grana cheese is a traditional Italian hard cheese, obtained from unpasteurized cow’s milk, aged for a minimum period of nine months up to four years. This type of cheese is of great importance for Italy’s food culture and economy; two brands of grana cheese, “Grana Padano” and “Parmigiano Reggiano,” are recognized with the Protected Designation of Origin certificate (PDO). Grana cheeses are high-quality manufactured products; after a 9-month storage period, the *a_w_* varies between 0.92 and 0.85 on the rind and between 0.94 and 0.87 if a thicker layer is considered; the relative humidity of storehouses ranges between 72% and 88% [1]. As defined in the guidelines of many PDO products, the ripening of hard cheese is carried out normally in a T range from 10–20 °C. For Italian grana cheese, the environmental conditions during aging are between 15 and 22 °C, as described in “Parmigiano Reggiano” and “Grana Padano” cheese production guidelines [2].

The development of filamentous fungi in grana cheese during ripening, limited to the rind surface, is common and accepted; in fact, growth of mold may be beneficial for the development of the characteristic flavor and aroma of dairy products, due to mold involvement with the degradation of lipids and protein. In addition, the surface microbiota can have a protective role against pathogenic spoilage microorganisms [3]. 

However, fungi belonging to *Aspergillus*, *Penicillium,* and *Mucor* spp. can cause cheese spoilage [4,5] with many undesired and adverse health effects, such as mycotoxin contamination. Therefore, during the ripening period, the cheese wheels are brushed at fixed times, to remove developing molds; further, the wheels are carefully brushed or washed before sale.

Globally, several studies have focused on the occurrence of mycotoxins in cheese; the most widely reported compounds were citrinin (CIT), penitrem A (PA), roquefortine C (ROQ-C), sterigmatocystin (STC), and ochratoxin A (OTA) [6,7,8,9,10,11]. *Penicillium* spp. is reported as the dominant fungal genus growing on cheese [4]. Information about penicillia growing on grana cheese during ripening has been reported by Decontardi et al. [10,12]. In particular, *P. crustosum* and *P. solitum* were the dominant species isolated, but other mycotoxin producing fungi identified were *A. flavus* and *P. verrucosum*. OTA was detected in all the cheese samples analyzed [10].

Although mycotoxin contamination may pose an important risk for consumer health and global trade [13], few studies have addressed the problem; therefore, the influence of ecological factors during cheese manufacturing and ripening on mycotoxin contamination has scarcely been investigated. The relevance of ecology in determining the dominant and/or the co-occurring contaminants has been stressed by several studies; therefore, mycotoxin prevalence is expected to be extremely variable in cheese ripening, depending on the storage conditions. A predictive model, accounting for the fungal infection cycle, is considered imperative to predict how ecological factors, such as temperature (T) and water activity (*a_w_*), affect fungal occurrence and mycotoxin contamination in matrices. So far, only limited attempts have been made to model the in vitro sporulation [14], growth, and mycotoxin production [1] of some mycotoxigenic fungi reported in grana cheese. Data on the impact of ecology on each step of the infection cycle are fundamental in order to develop predictive models for the risk of mycotoxin contamination during grana cheese ripening as a valuable support in risk assessments and management.

The aim of this study was to investigate: (i) the effect of T on growth and mycotoxin production by *A. versicolor*, *P. crustosum*, *P. nordicum*, *P. roqueforti,* and *P. verrucosum* artificially inoculated on grana cheese rind blocks; (ii) the partitioning of mycotoxins between the cheese rind and fungal mycelium; and (iii) the reliability of growth and mycotoxin production models, previously developed using data collected in in vitro trials with fungi inoculated on artificial media.

## 2. Materials and Methods

### 2.1. Cheese Blocks Sampling and Disinfection

Squared cheese rind blocks (Consorzio per la tutela del Formaggio Grana Padano, Lodi, Italy and Consorzio del Formaggio Parmigiano Reggiano, Reggio Emilia, Italy) of about 4 × 4 × 2 cm (approximate weight: 40 g) were collected from the flat, upper part of 12-month old ripening grana cheese wheels, vacuum-packed in special plastic bags and stored at 5 °C until use. The water activity (*a_w_*) of the matrix was measured using an Aqualab LITE device (version 1.3 © Decagon devices Inc., Pullman, WA, USA). 

The cheese rind blocks were disinfected as described by Battilani et al. [15] with minor changes, and all operations were performed under a sterile hood. In brief, the cheese blocks were washed in a 2% solution of NaClO (Carlo Erba, Milan, Italy) for two minutes, washed in absolute ethanol for two minutes and dried for about five minutes, and passed over a Bunsen burner flame three times for 2 s.

### 2.2. Inoculum Preparation, Inoculation, and Incubation of the Cheese Blocks

Ingredients for media were purchased from Himedia Laboratories (Mumbai, India). Fungal strains of five mycotoxin producing fungi originating from various official fungal collections (Table 1) were centrally inoculated on 60 mm diameter plates filled with Malt Extract Agar (MEA [16]) and incubated in the dark at 25 °C for seven days.

Fungi inoculation was performed using the 7-day old colonies. For each fungal strain, a small amount of mycelium and spores was collected using a sterile metal needle and centrally laid on the external surface of the cheese blocks. The trial was managed in triplicates [15]. The amount of inoculum was quantified by picking a similar quantity of fungal material used as inoculum, with 1 mL of bidistilled water added and counting the number of spores using a haemocytometer. The inoculum concentration assessment was repeated 3 times for each fungal isolate.

Humid chambers were prepared using round glass cups (diameter: 19 cm; height: 9 cm; approximate volume: 2.350 L) with 100 mL of a sterilized NaCl (Carlo Erba, Milan, Italy) solution added, prepared as described by Dallyn and Fox [17] to obtain 0.93 *a_w_*. A properly shaped metal grid was sterilized and placed inside the cups. The inoculated cheese blocks were then put on the grid to avoid any contact with the salt solution [18]. All the cups were then sealed with a plastic film and incubated at temperatures (T) ranging from 10 to 30 °C (5 °C step) with a 12 h photoperiod for 21 days.

### 2.3. Data Collection

Fungal growth: at the end of incubation, two perpendicular diameters of the fungal colony (mm) were measured. Then, the colonies were removed, by gently scraping the mycelium from the cheese surface, and put in a Falcon^®^ plastic vial with a screw cap. Both the Falcon vials and the cheese blocks were stored at −20 °C until use.

Mycotoxin Analysis: the chemicals and solvents used for the extraction and clean-up solutions were ACS grade or equivalent (Carlo Erba, Milan, Italy), mycotoxin standards were obtained from Sigma-Aldrich (St. Louis, MO, USA). Mycotoxin analyses were performed separately both on the mycelium colonies and the cheese blocks. Rind cheese and fungal mycelium were ground and homogenised before analysis. All samples were subdivided for consistency in three aliquots for mycotoxin extraction using CH_3_CN:H_2_O (80:20 *v/v*) for MPA, PR-toxin, ROQ-C, STC and PA, CH_3_CN:10 mM H_3_PO_4_ (70:30 *v/v*) for CIT and CH_3_OH:3% NaHCO_3_ (50:50 *v/v*) for OTA. Toxin analyses were performed by HPLC-MS/MS (Thermo-Fisher Scientific, San Jose, CA, USA) and standards for STC, CIT, ROQ, MPA, OTA, PR toxin, and PA were prepared, as described by Camardo Leggieri et al. [1]. Data were reported as total ng of mycotoxin on cheese block or mycelium. Limits of detection (LODs) were 1 ng for ROQ-C, 5 ng for MPA, PA, STC, OTA, and CIT, 10 ng for PR-toxin.

### 2.4. Data Analysis

Statistical analyses were performed using SPSS v.25 (SPSS Inc., Armonk, NY, USA, 2012) software. Mycotoxin production data were ln transformed to homogenize the variance and tested for normality using the Shapiro-Wilk test. As all data succeeded the normality test, the one way-ANOVA was applied. The Tukey test was applied to highlight significant differences between means.

Modelling of growth data: data of fungal growth on natural substrate, intended as the diameter of fungal colonies grown on cheese rind, at different T regimes, were rated on the maximum value observed to obtain the relative growth on a 0–1 scale (0 = no growth; 1 = maximum growth). Then, fungal relative growth on the cheese rind was fitted to the models published by Camardo Leggieri et al. [1]. Briefly, Bete function [19] and a logistic function described in vitro fungal growth on artificial medium (Czapeck Yeast Agar (CYA)) under different T and *a_w_* regimes, respectively. Residuals were calculated as the mathematical difference between relative growth collected on natural substrate and the function. Positive values for the residuals (on the y-axis) mean the prediction was overestimated, negative values mean the prediction was underestimated and 0 means the estimation was correct. 

## 3. Results

The fungal inoculum concentration, measured on three replicates prepared for each fungus, was ~10^5^ spore/mL; therefore, this was assumed as the number of spores distributed on each cheese block. 

### 3.1. Fungal Growth

Almost all fungi included in the study grew in the T range considered (10–30 °C). *A. versicolor* and *P. roqueforti* did not grow at 10 °C while *P. nordicum* and *P. roqueforti* did not grow at 30 °C; for these aforementioned combinations fungus × temperature, at the end of incubation the diameter of the fungal colony was comparable to the initial inoculum. Therefore, no measurable growth was assumed for *A. versicolor* and *P. roqueforti* at 10 °C and for *P. nordicum* and *P. roqueforti* at 30 °C.

**Figure 1:**
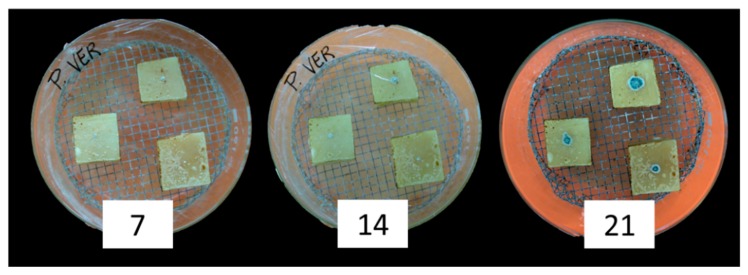
Growth of *Penicillium verrucosum* inoculated on cheese blocks (triplicate) after 7, 14, and 21 days of incubation at 25 °C with 0.93 water activity (*a_w_*).

All the T regimes tested significantly affected mean fungal growth (*p* ≤ 0.01), with maximum colony diameter at 20 °C and minimum at 10 and 30 °C (Table 2). Significant differences between the two fungal genera included in the study were detected, with 8.5 versus 13.5 mm mean colony diameter, respectively, for *A. versicolor* and *Penicillium* spp. (Table 2). Almost all the fungal colonies showed the production of spores after 10 days of incubation; colonies of *P. verrucosum* are shown in Figure 1, as an example.

**Figure 2:**
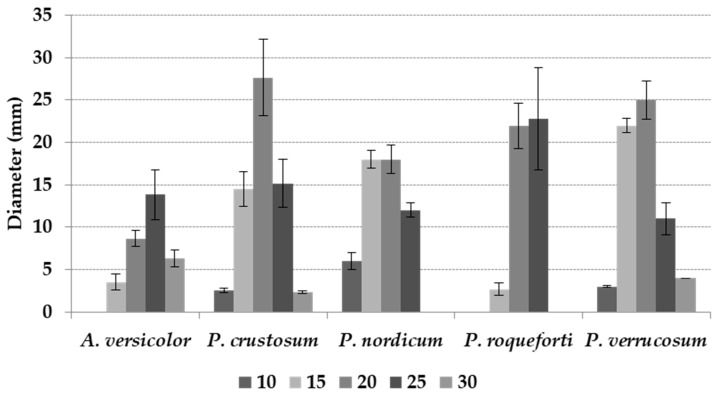
Mean colony diameter (mm) after 21 days of incubation of *Aspergillus versicolor*, *Penicillium crustosum*, *P. nordicum*, *P. roqueforti,* and *P. verrucosum* at different temperatures (T) of incubation (10–30 °C, 5 °C step). The bars indicate the mean standard error.

The interaction between T and fungal species was significant (*p* ≤ 0.01). *A. versicolor* and *P. crustosum* showed optimal growth at 25 and 20 °C, respectively; both *P. nordicum*, and *P. verrucosum* grew optimally at 15 and 20 °C, while *P. roqueforti* at 20 and 25 °C (Figure 2).

Fungal growth was commonly very limited in the marginal T regimes studied. In the respective optimal T regime, after 21 days of incubation (Figure 2), *P. crustosum* reached the largest colony diameter (28 mm), followed by *P. verrucosum* (25 mm), *P. roqueforti* (23 mm), and *P. nordicum* and *A. versicolor* (about 14 mm each). Colony diameters in detail for every species are reported in Table 2.

### 3.2. Mycotoxin Producion

**Figure 3:**
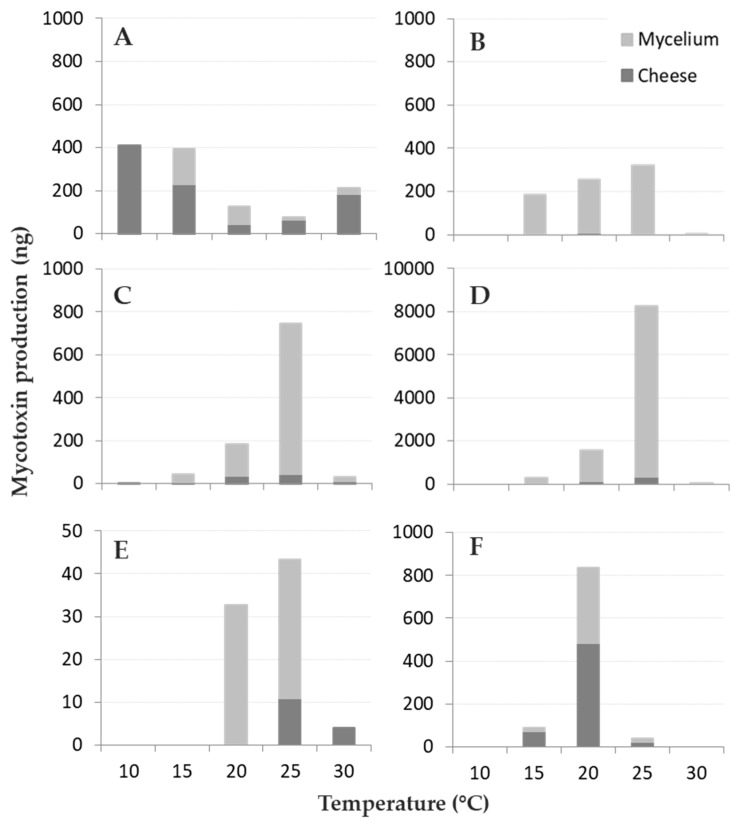
Mycotoxin (ng) partitioning between cheese rind and fungal mycelium for the fungi considered at different temperature levels (10–30 °C, step 5 °C): (**A**) sterigmatocystin (STC)–*Aspergillus versicolor*, (**B**) penitrem A (PA)–*Penicillium crustosum*, (**C**) roquefortine C (ROQ-C)–*P. roqueforti*, (**D**) ROQ-C–*P. crustosum* (**E**) ochratoxin A (OTA)–*P. nordicum* and (**F**) OTA–*P. verrucosum*. The amount of mycotoxin detected in cheese rind is reported in dark grey, while the pale grey part of the bars represents the number of mycotoxins detected in fungal mycelium.

All the toxins reported in the literature for the studied fungi were detected, with the exception of MPA, Pr-Tox, and CIT, produced by *P. roqueforti* and *P. verrucosum*, respectively; these toxins were always below the LOD (Figure 3).

The ANOVA was applied to all the data on mycotoxins produced after 21 days of incubation on the cheese blocks, adding up the toxins detected in the fungal colony and on the cheese blocks (Table 3). T had a significant impact on all the mycotoxins analysed, as with the considered partitioning (rind and colony). The interaction “T” × “Partitioning” resulted significant only for PA and OTA produced by *P. crustosum* and *P. nordicum*, respectively (Table 3). 

All fungi produced their respective mycotoxins between 15 and 25 °C (Table S1 and Table 3); in particular, *A. versicolor* and *P. roqueforti* produced mycotoxins in the whole T range tested (10–30 °C), while *P. nordicum* only at between 20 and 30 °C. At the lowest T tested (10 °C) only *A. versicolor* produced the highest amount of STC detected, while the minimum was measured after incubation at 25 °C. On the contrary, all the other fungi showed an opposite trend, with the optimal T for mycotoxin production at 20–25 °C, and 20 °C for OTA production by *P. verrucosum*. Therefore, four out of five species followed a “bell-shaped” trend in producing their mycotoxins in different T regimes, *A. versicolor* showed a reverse bell, with a remarkable decrease in STC production at 20 and 25 °C compared to 10 and 15 °C. 

The interaction between T and partitioning between the cheese rinds and fungal mycelium is reported in Figure 3, even if not statistically significant. Mycotoxin contamination in cheese rind was generally limited: in three out of five fungal species (*P. crustosum*, *P. nordicum,* and *P. roqueforti*) the majority of the mycotoxins produced remained located in the fungal colony (~90%) while the opposite happened for *A. versicolor* and *P. verrucosum* (Figure 3A,F) with 71% and 58% of STC and OTA detected in cheese rind, respectively. For *A. versicolor* (Figure 3A), 85% of STC was recovered in cheese rind at the highest T tested while the lowest (34%) was detected at 20 °C. Regarding OTA, 81% was released in cheese by *P. verrucosum* at 15 °C, around 55%–58% at 20 and 25 °C (Figure 3F) and only 25% at 25 °C was released by *P. nordicum* in cheese. 

### 3.3. Modeling Fungal Growth

**Figure 4:**
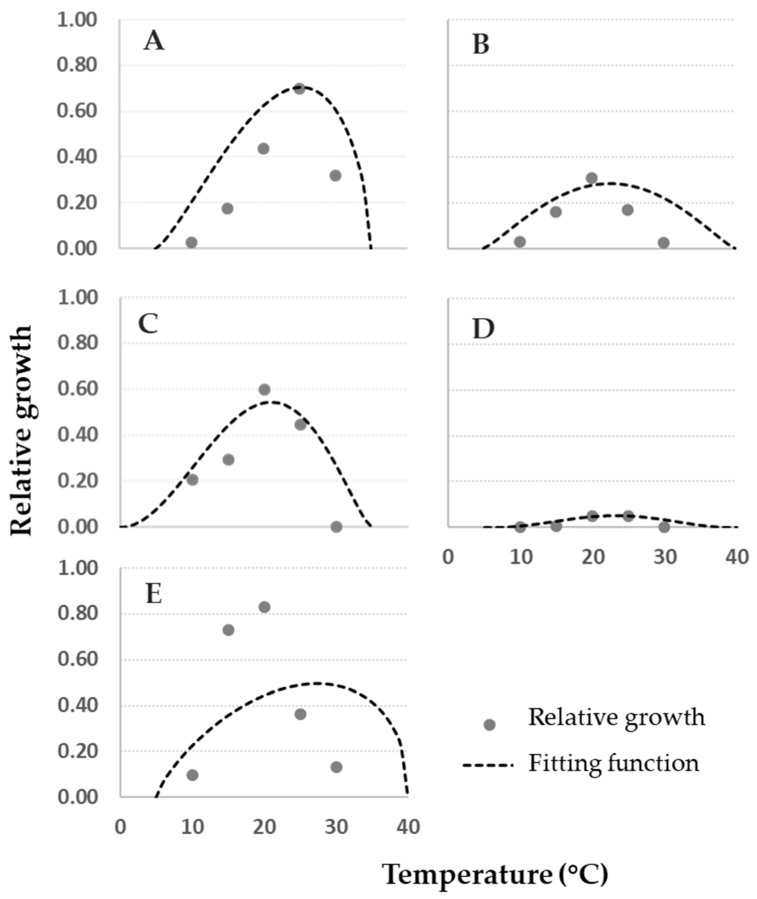
Data of fungal relative growth on cheese rind (grey dots) and the fitting functions based on relative growth data obtained on Czapeck Yeast Agar (dashed lines, [1]) for (**A**) *Aspergillus versicolor*, (**B**) *Penicillium crustosum*, (**C**) *P. nordicum*, (**D**) *P. roqueforti,* and (**E**) *P. verrucosum* at different temperature regimes (0–40 °C, step 5 °C).

Data on fungal relative growth on cheese rind obtained in this study were compared with modeled data of fungal growth in vitro at 0.93 *a_w_* [1]. In Figure 4, plotted data of cheese rind (grey dots) vs. artificial medium (dashed lines) commonly followed a comparable trend.

**Figure 5:**
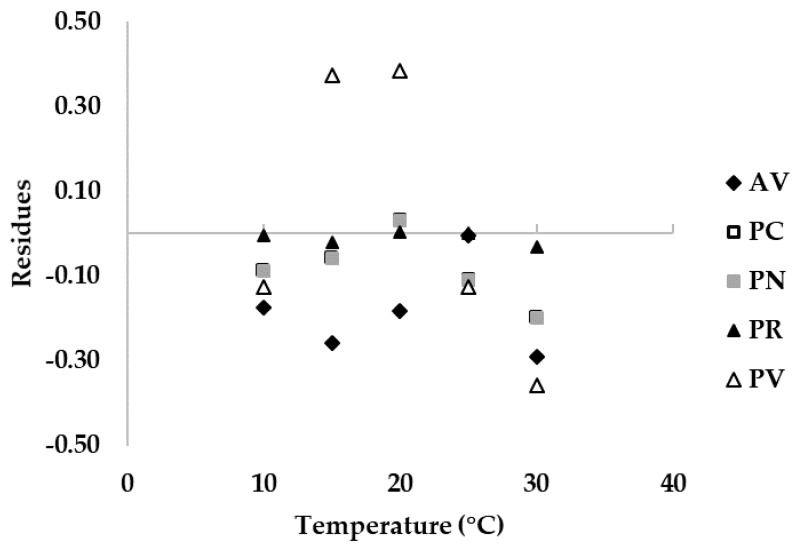
Plot of residuals between fungal relative growth on cheese rind and the fitting functions based on relative growth data obtained on Czapeck Yeast Agar, calculated for *Aspergillus versicolor* (AV), *Penicillium crustosum* (PC), *P. nordicum* (PN), *P. roqueforti* (PR), and *P. verrucosum* (PV) at different incubation temperatures (0–40 °C, step 5 °C).

Detailed information on the comparison between growth on cheese rind and the model built based on in vitro data are shown in Figure 5, where residuals were calculated. In particular, with the exception of *P. verrucosum* (Figure 4E), all the cheese rind data can be well-described by the functions developed with data collected in vitro, as points are close to the lines (Figure 4). Trends described by cheese rind data are narrower than the fungal relative growth models, in particular for *A. versicolor*, *P. crustosum,* and *P. nordicum,* nearly overlapping at the optimal T of 25 °C. For *P. verrucosum* (Figure 4E) at T = 15–20 °C, a higher fungal relative growth on cheese rind was observed compared to the model, which corresponds to an under-estimation of fungal growth on cheese rind by the fitting function and to a positive residue score (Figure 5). In general, with the exception of *P. verrucosum*, residues are close to 0 for optimal growth and for a broad range of T.

### 3.4. Modeling Mycotoxin Production

As well as for fungal growth and for mycotoxin production, data on cheese rind obtained in this study were compared with modeled data of mycotoxin production in vitro [1]. In this study, toxins were produced on cheese in a very narrow range, with comparable amounts only in two T regimes and 10 times lower amounts both for lower or higher T. Therefore, it was not possible to fit data on previously developed functions, neither to develop new functions. The optimal T for toxin production has been confirmed for all fungi.

## 4. Discussion

The co-occurrence of toxigenic fungi on grana cheese, during ripening and storage [5,10,20] is a matter of concern for manufacturers and consumers, because of the possible production of different mycotoxins. There is a complete lack of regulation for mycotoxins (with the exception of aflatoxin M_1_ arising from milk used for cheese production) in cheese. OTA has been reported in grated cheese [21] and is regulated for several commodities [22,23]. Despite the regulations in force, OTA has been reported in human plasma and breast milk [24,25,26,27,28], confirming human exposure to OTA, in particular of vulnerable groups of consumers such as infants.

All the mycotoxins considered in this study could theoretically co-occur in cheese. The co-occurrence of toxic compounds in feed and food is of increasing interest in the scientific community [29,30,31]. The knowledge available is still limited, but it suggests synergistic effects of mycotoxin mixtures, with an impact on consumer health possibly more severe compared to the addition of single mycotoxin effects [32,33].

Cheese consumption worldwide is an important component of human diets [34,35] and it is therefore essential to understand the conditions that favor mycotoxin production and, possibly, to predict their occurrences.

*Penicillium* species are commonly reported as the dominant contaminant fungi in cheese [4] and of these *P. roqueforti*, *P. crustosum* [36], and *P. citrinum* are the most significant [37]. *P. nordicum* and its closely related species *P. verrucosum* are also signaled, even if more reported in ripened pork meat [15,18,36]. *A. versicolor* was also included because it has previously been reported as a cheese contaminant [4].

Almost all the fungi were able to grow on the real matrix cheese. Despite a few differences, the impact of T is consistent with previous reports, suggesting that the fungi included in this study grow readily between 15 and 25 °C on different media culture [1,38,39]. A matrix effect was noticed for *P. verrucosum*; in fact, the fitting to the model developed with data collected on CYA did not fit perfectly, with growth enhanced on cheese at 15 and 20 °C. As for growth, also mycotoxin production is dependent on the growth media and environmental conditions. 

The matrix effect is widely reported in literature, with emphasis on mycotoxin production, e.g., for *P. nordicum* and *P. verrucosum* [18,40]. The nutritional matrix effect was recently pointed out also by Coton et al. [41]; a strain of *P. nordicum* produced OTA and CIT in Comté cheese, while these metabolites were not detected in yeast extract with supplements medium (YES). *P. crustosum* was reported to produce ROQ-C on several substrates, but the highest amount was detected on cheese analogue media [42]. In the present study, *P. crustosum* produced more ROQ-C (~10 times fold) than *P. roqueforti* in all the tested conditions; this is in agreement with in vitro studies, but the difference in toxin production between the two species was limited, around two- to four-fold. Further, Kokkonen et al. [42] reported a remarkable amount of CIT produced by a strain of *P. verrucosum* on bread analogue media, although not detected (<LOD) on cheese.

All the toxins analyzed in this study were detected, except MPA, Pr-Tox, and CIT produced by *P. roqueforti* and *P. verrucosum* (below the LOD); therefore, mycotoxin synthesis is possible during grana cheese ripening. T range for toxin production was between 15 and 25 °C, in agreement with the findings of Sànchez-Monter at al. [40]; they reported that *P. verrucosum* and *P. nordicum* produced OTA readily in the range 15–25 °C on dry-cured meat products. On the contrary, STC was optimally produced by *A. versicolor* at 10–15 °C; this fungus is known as mesophilic, with a minimum T for growth of 9 °C at 0.97 *a*_w_ [43].

Both the limited T regimes that enabled toxin production and the anomalous behavior of *A. versicolor* justify the difficulties of transferring the predictive models obtained in vitro for mycotoxin production to real conditions on cheese. In addition, a previous study carried out on dry-cured ham considered incubation periods of *P. nordicum* on ham tassels up to 190 days, on account of the long ripening period of the product (minimum 12 months); a considerable impact of long incubation time on OTA production was detected [18]. Only in one study, regarding CIT and OTA production by *P. verrucosum* in a semi-hard French PDO cheese, longer incubation periods (42 days) compared to the 21 days of this study were tested [41]; at 20 °C both mycotoxins were produced after seven days, but for a longer incubation period (35 and 42 days) OTA production was higher than CIT and after 42 days OTA concentration reached its highest point. Therefore, an increase of the incubation period could be useful to better simulate the real ripening period (months), to give fungi enough time to adapt to the matrix and optimize their metabolism on the nutritional source. Data coming from that kind of study should be suitable to test the existing models or eventually to develop new ones.

The partitioning of mycotoxins between the mycelium and their release on cheese rind varied significantly for different fungi, irrespective of incubation T. In particular, the occurrence of STC and OTA produced by *A. versicolor* and *P. verrucosum*, respectively, was the highest in cheese rind; for all the other combinations, almost all the toxins were not released on the medium. This finding is crucial in practice; in fact, the removal of fungal mycelium should guarantee a safe product, at least for most of the toxins considered. However, studies with longer incubation periods should be carried out to confirm this result. In agreement with our results, Coton at al. [41] detected on Comté cheese OTA and CIT, produced by *P. verrucosum*, up to 1.6 cm in depth, depending on the incubation time; additionally, they underlined that the highest concentration was detected when the mold was present. The rind of grana type cheeses is edible and a standard amount of rind is permitted in grated cheese; therefore, knowledge of both the ecological needs of toxin-producing fungi and the rate of mycotoxin release on the matrix are important for the prevention of consumer exposure.

On the other hand, this should also support grana cheese rind management during ripening. In fact, during ripening, cheese wheels repeatedly undergo surface clean-up brushing operations. The growing mold is therefore removed, but fungal spores are also airborne-spread, increasing their dispersal in the storehouse and providing further opportunities of growth due to the micro-damage caused on the cheese rind surface. Camardo Leggieri et al. [14], reported that *P. crustosum*, *P. nordicum,* and *P. verrucosum* had shown an optimum T for sporulation between 20 and 25 °C and their sporulation continued up to *a_w_* = 0.87 (*a_w_* = 0.83 for *P. nordicum*); therefore, they are adapted to the environmental conditions of storehouses for grana cheese ripening. A high spore concentration is expected when fungi grow on cheese rind, as confirmed by this study, with sporulation reported for all fungi from 10 days of incubation.

In conclusion, the knowledge acquired in this study confirms that the (co-)occurrence of several mycotoxin in cheese is possible. Some fungi release most of the toxins in the medium and neither T during ripening nor cheese *a_w_* can act as limiting factors; this should be considered in the grana cheese management, even if no regulation is actually in force. Further studies, following the same approach reported here, but with longer incubation periods, should be planned in the future to make results more consistent and to support mycotoxin modelling. Nevertheless, these outcomes are important and may contribute to the optimization of grana cheese management.

## Figures and Tables

**Table 1:** Fungal strains used in this study, with the country, matrix of isolation, and mycotoxins reported in literature for these fungal species.

Species	Strain Code ^a^	Country of Isolation	Matrix of Isolation	Mycotoxin
*A. versicolor* ^b^	CBS 108959	Denmark		Sterigmatocystin (STC)
*P. crustosum*	CBS 115503	The Netherlands		Penitrem A (PA) Roquefortine C (ROQ-C)
*P. nordicum*	BFE 856	Italy	Ham	Ochratoxin A (OTA)
*P. roqueforti*	CBS 221.30	USA		Roquefortine C (ROQ-C) Mycophenolic acid (MPA) Pr-Toxin (PR-Tox)
*P. verrucosum*	MUM 16.76	Italy	Cheese	Ochratoxin A (OTA) Citrinin (CIT)

^a^ CBS: Westerdijk Fungal Biodiversity Institute; MUM: Micoteca da Universidade do Minho; BFE: Federal Research Centre for Nutrition and Food. ^b^ *A. versicolor* CBS 108959 was later identified as *A. jensenii* by Decontardi et al. [12] but *A. versicolor* will be kept in the text to remain consistent with the CBS website database labelling.

**Table 2:** Analysis of variance (ANOVA) for fungal growth (colony diameter; mm after 21 days of incubation) in different regimes of temperature (10–30 °C, step 5 °C) and for diverse fungi (*Aspergillus versicolor*, *Penicillium crustosum*, *P. nordicum*, *P. roqueforti,* and *P. verrucosum*). Standard deviation of growth was reported in parenthesis.

Factors	Growth	
**Temperature (°C) (A)**	**	
10	3.4	*d* *^§^*
15	11.5	*c*
20	22.1	*a*
25	16.3	*b*
30	3.1	*d*
**Fungi (B)**	**	
*A. versicolor*	8.5	*b*
*P. crustosum*	13.1	*a*
*P. nordicum*	13.4	*a*
*P. roqueforti*	13.3	*a*
*P. verrucosum*	14.1	*a*
**(A × B)**	**	

*^§^* Different letters define significant difference according to the Tukey test; ** *p* ≤ 0.01.

**Table 3:** Analysis of variance (ANOVA) for mycotoxin production (ng) in different regimes of temperature (10–30 °C, step 5 °C) and for different partitioning (cheese rind or mycelium). Standard deviation of mycotoxin were reported in parenthesis.

Factors	*A. versicolor*		*P. crustosum*				*P. nordicum*		*P. roqueforti*		*P. verrucosum*	
	STC		PA		ROQ-C		OTA		ROQ-C		OTA	
**Temperature (°C) (A)**	**		**		**		**		**		**	
10	409.3	a *^§^*	<LOD	b	<LOD	c	<LOD	c	3.8	c	<LOD	c
	(±1.84)								(±1.13)			
15	197.3	ab	92.6	a	151.8	b	<LOD	c	19.4	bc	43.2	b
	(±1.60)		(±12.45)		(±5.14)				(±2.03)		(±2.93)	
20	64	bc	125.4	a	787.4	a	16.4	ab	91.7	ab	418.6	a
	(±2.25)		(±8.21)		(±3.75)		(±5.63)		(±2.48)		(±2.63)	
25	38.8	bc	160	a	4140.5	a	21.6	a	370.5	a	12.4	bc
	(±2.96)		(±14.71)		(±5.63)		(±3.75)		(±5.19)		(±4.49)	
30	122.2	c	<LOD	b	22.3	b	4.0	bc	14.7	c	<LOD	c
	(±2.67)				(±2.84)		(±3.35)		(±1.73)			
**Partitioning (B)**	**		**		**		**		**		n.s.	
Cheese rind	186.8	a	1.5	b	101.9	b	3.0	b	19.0	b	113.5	a
	(±2.64)		(±2.31)		(±8.07)		(±3.00)		(±2.38)		(±11.25)	
Mycelium	76.1	b	204.4	a	2642	a	16.3	a	266.9	a	81.6	b
	(±3.39)		(±6.02)		(±6.03)		(±5.58)		(±3.81)		(±9.21)	
**A × B**	n.s		**		n.s.		**		n.s.		n.s.	

*^§^* Different letters define significant difference according to the Tukey test; n.s.: not significant; ** *p* ≤ 0.01.

## Supplementary Materials

The following are available online at https://www.mdpi.com/2076-2607/8/1/69/s1, Table S1: Colony diameters (mm) and mycotoxin production (ng) after 21 days of incubation. Partitioning levels of mycotoxin produce by the fungi on cheese rind and by fungal mycelium were provided, as well as the total amount.
